# 2-hydroxyethyl methacrylate-derived reactive oxygen species stimulate ATP release via TRPA1 in human dental pulp cells

**DOI:** 10.1038/s41598-022-16559-8

**Published:** 2022-07-19

**Authors:** Ai Orimoto, Chiaki Kitamura, Kentaro Ono

**Affiliations:** 1 Division of Endodontics and Restorative Dentistry, Kyushu Dental University, 2-6-1 Manazuru, Kokurakita-ku, Kitakyushu, Fukuoka 803-8580 Japan; 2 Division of Physiology, Kyushu Dental University, 2-6-1 Manazuru, Kokurakita-ku, Kitakyushu, Fukuoka 803-8580 Japan

**Keywords:** Analytical biochemistry, Biophysical methods

## Abstract

Extracellular ATP (adenosine triphosphate) and transient receptor potential ankyrin 1 (TRPA1) channels are involved in calcium signaling in odontoblasts and dental pain. The resin monomer 2-hydroxyethyl methacrylate (HEMA), used in dental restorative procedures, is related to apoptotic cell death via oxidative stress. Although the TRPA1 channel is highly sensitive to reactive oxygen species (ROS), the effect of HEMA-induced ROS on ATP release to the extracellular space and the TRPA1 channel has not been clarified in human dental pulp. In this study, we investigated the extracellular ATP signaling and TRPA1 activation by HEMA-derived ROS in immortalized human dental pulp cells (hDPSC-K4DT). Among the ROS-sensitive TRP channels, TRPA1 expression was highest in undifferentiated hDPSC-K4DT cells, and its expression levels were further enhanced by osteogenic differentiation. In differentiated hDPSC-K4DT cells, 30 mM HEMA increased intracellular ROS production and ATP release, although 3 mM HEMA had no effect. Pretreatment with the free radical scavenger PBN (N-tert-butyl-α-phenylnitrone) or TRPA1 antagonist HC-030031 suppressed HEMA-induced responses. These results suggest that ROS production induced by a higher dose of HEMA activates the TRPA1 channel in human dental pulp cells, leading to ATP release. These findings may contribute to the understanding of the molecular and cellular pathogenesis of tertiary dentin formation and pain in response to dental biomaterials.

## Introduction

Acrylic monomers routinely used in dentistry have been known to cause allergic reactions in dental patients^1,2^. The release of acrylic monomers from bond-resin-based dental restorative materials causes local and systemic allergic reactions^3,4^. The monomer 2-hydroxyethyl methacrylate (HEMA) is a water-soluble acrylic resin monomer, is used in dental restorative procedures such as repair or restoration and root canal sealers, and is one of the main components in dentin bonding resins with contents varying from 30 to 55%^5^. HEMA can easily diffuse from polymer-based restorative materials^6^. As HEMA can leach out through dentin tubules into the pulp tissue, it may cause pulpal inflammation, resulting in the generation of pain impulses^7^.

Numerous studies have reported HEMA-induced cytotoxicity in various cell types, including dental pulp cells^8^. HEMA-derived reactive oxygen species (ROS) have been suggested as the main cytotoxic factors^9^. ROS production is attributed to HEMA-induced formation of mitochondrial superoxide anions^10^. The expression of antioxidant enzymes, including heme oxygenase (HO-1), following ROS production, protects the cells against HEMA^11,12^. *HO-1*, a major downstream gene of the Nrf2-ARE (nuclear factor erythroid 2-related factor/antioxidant response element) pathway, is highly upregulated to attenuate cellular oxidative stress, preventing apoptosis and promoting cell survival^13–17^.

Transient receptor potential (TRP) channels respond to various physical, thermal, and chemical stimuli^18,19^. TRPA1, TRPV1, and TRPV4 are known as oxidant-sensitive TRP channels, and TRPA1 has the highest oxidation sensitivity among the TRP channels^20^. The channel is predominantly expressed in human dental pulp, and its expression is significantly higher in painful pulp than in normal pulp^21^. Recent studies have reported that TRPA1 expression is upregulated in odontoblasts aligned along tertiary dentin formed under carious lesions^22,23^. Tertiary dentin is produced by odontoblasts in response to external stimuli. It contains entrapped odontoblasts originating from undifferentiated precursors, including dental pulp stem cells (DPSCs), in response to dentinal tubules-derived stimulation^24,25^. Application of dentin bonding systems to seal both dentin and pulp may affect tertiary dentin formation^26^. However, it is not known whether application of dentin bonding systems and their by-products induce TRPA1 activation.

Extracellular adenosine triphosphate (ATP) is thought to act as a neurotransmitter and neuromodulator, and is considered a powerful algogenic substance^27,28^. Recent studies have reported that human odontoblast-like cells can release ATP via pannexin-1 (PANX1), the gap junction hemichannel protein, in response to TRPA1 activation. Consequently, released ATP activates P2X3 receptors on pulpal neurons, which promotes the extracellular calcium influx, suggesting that ATP release from odontoblasts following TRP channel–dependent calcium influx functions as a neurotransmitter to drive the sensory transduction mechanism of dentinal sensitivity^29^. These findings suggest that odontoblastic/osteoblastic differentiated dental pulp stem cells are more suitable for understanding cellular responses in dental pulp than immature cells. We previously established immortalized dental pulp stem cells (DPSCs) expressing R24C mutant cyclin-dependent kinase 4 (CDK4^R24C^), Cyclin D1, and telomerase reverse transcriptase (TERT), which were termed hDPSC-K4DT cells from the last characters of the introduced genes (CDK4, Cyclin D1, TERT)^30^. The hDPSC-K4DT cells have the advantage of maintaining their original chromosomal pattern and stemness characteristics, suggesting that they can be a useful tool with relatively intact characteristics that mimic primary human dental pulp stem cells.

In the present study, we investigated the cytotoxicity of ROS generated following treatment of hDPSC-K4DT cells with different HEMA concentrations and examined whether it is involved in TRPA1-dependent ATP release, which would contribute to pain response to stimuli, such as exposure of dental pulp to chemicals.

## Materials and methods

### Cell culture and differentiation

HDPSC-K4DT cells, immortalized DPSCs, were previously established^30^ and cultured in high-glucose Dulbecco’s minimum essential medium (FUJIFILM Wako Chemicals, Miyazaki, Japan) containing 10% heat-inactivated fetal bovine serum (FBS; Sigma-Aldrich, St. Louis, MO, USA) and 1% penicillin–streptomycin solution (FUJIFILM Wako Chemicals) at 37 °C in a humidified atmosphere containing 5% CO_2_. As a control for hDPSC-K4DT cells, the human cementoblast cell line (HCEM), kindly provided by Dr. Takada^31^, was cultured under similar conditions. We used HCEM cells as the negative control cell line of the expression of TRP channels. Osteogenic differentiation of hDPSC-K4DT cells was performed using osteoinduction medium (Osteoblast-Inducer Reagent, Takara, Shiga Japan) for 12–15 days, according to the manufacturer’s instructions. When the cultured cells reached 70% confluence, the normal culture medium was replaced with osteogenic induction medium. Undifferentiated and differentiated hDPSC-K4DT cells after drug treatment were subjected to quantitative reverse transcriptase-polymerase chain reaction (RT-qPCR) analysis, ROS detection assay, and ATP release assay.

### Drug treatments

To determine the involvement of ROS and TRPA1 in HEMA-induced responses, the culture medium of the cells seeded into 6 or 96-well culture plates was replaced with or without the free radical scavenger PBN (N-tert-butyl-α-phenylnitrone; Sigma-Aldrich) at 6 mM and the TRPA1-selective antagonist HC-030031 (FUJIFILM Wako) at 30 or 100 μM, which were dissolved directly in D-MEM supplemented with 10% FBS. After the cells were preincubated at 37 °C for 1 h under 5% CO_2_ with or without PBN and HC-030031, the medium containing HEMA (Kanto Chemical, Tokyo, Japan) was added into each well at 0–30 mM. We directly diluted neat HEMA with the culture medium immediately before use and added the resultant medium to the wells. The cells were then incubated at 37 °C for an additional 1–24 h under 5% CO_2_.

### Cell viability analysis

To assess the effect of HEMA on the viability of hDPSC-K4DT cells, we used the Cell Counting Kit-8 (CCK-8; Dojindo Molecular Technologies, Gaithersburg, MD, USA). HDPSC-K4DT cells were seeded in 96-well culture plates at a density of 2.5 × 10^3^ cells/well, incubated at 37 °C for 24 h, and treated with HEMA (Kanto Chemical) at the indicated concentrations for 24 h. Then, 10 μl of the Cell Counting Kit-8 solution containing tetrazolium salt (WST-8) was added to the cell culture medium and incubated at 37 °C for 1 h. Absorbance at 450 nm was measured using a microplate reader (Bio-Rad Laboratories, Tokyo, Japan).

### RT-qPCR analysis

For preparation of total RNAs, hDPSC-K4DT cells were seeded into 6-well culture plates at a density of 1 × 10^5^ cells/well. The total RNA was extracted from the collected cells using a NucleoSpin RNA kit (Takara Bio, Shiga, Japan), and cDNA was synthesized using the iScript cDNA Synthesis Kit (Bio-Rad Laboratories, Tokyo, Japan). cDNA (4 ng) was amplified by qPCR using Fast SYBR™ Green Master Mix (Applied Biosystems, Thermo Fisher Scientific, Tokyo, Japan) and specific primer sets for human genes and QuantStudio 3 PCR system (Applied Biosystems, Thermo Fisher Scientific, Tokyo, Japan). The following specific primer sets were used in this study for human genes: transient receptor potential ankyrin 1 (TRPA1), 5′- TGGACACCTTCTTCTTGCATT-3′ and 5′-TCATCCATTTCATGCAGCAC-3′; TRPV1, 5’-CTACAGCAGCAGCGAGACC-3′ and 5′-CCTGCAGGAGTCGGTTCA-3′; TRPV4, 5′-CAACAACGACGGCCTCT-3′ and 5′-GGATGATGTGCTGAAAGATCC-3′; alkaline phosphatase (ALP), 5′-ATGCTGAGTGACACAGACAAGAAG-3′ and 5′-GGTAGTTGTTGTGAGCATAGTCCAC-3′; heme oxygenase (HO-1), 5′-AGAGAATGCTGAGTTCATGAGGA-3′ and CAGCTCTTCTGGGAAGTAGACAG- 3′; collagen type I α 1 chain (COL1A1), 5′-TGAAGGGACACAGAGGTTTCA-3′ and 5′-ACCATCATTTCCACGAGCA- 3’; GAPDH, 5′-AGCTCACTGGCATGGCCTTC-3′ and 5′-TCACACCCATGACGAACATGG-3′. Gene expression values were calculated based on the ΔΔCt method, using GAPDH as an internal control to normalize mRNA levels.

### ROS detection

To determine the levels of intracellular ROS in hDPSC-K4DT cells treated with HEMA, we used the ROS Detection Cell-Based Assay Kit (DCFDA) (Cayman, Ann Arbor, MI, USA), according to the manufacturer’s protocol. In brief, hDPSC-K4DT cells that are immortalized DPSCs previously established^30^ were seeded in a black tissue culture-treated 96-well plate at a density of 5.0 × 10^3^ cells/well, cultured to 70% confluence, and incubated at 37 °C in osteogenic induction medium for 12 days that was found the induction duration with the upregulated TRPA1 mRNAs levels. For the reactive oxygen species (ROS) detection assay, hDPSC-K4DT cells were pretreated with 6 mM PBN (N-tert-butyl-α-phenylnitrone) for 1 h, then stimulated with HEMA (Kanto Chemical, Tokyo, Japan) at 3 or 30 mM for an additional 3 h at 37 °C in a humidified 5% CO_2_ atmosphere. Then, the medium was aspirated, the cells were rinsed with 150 μl of cell-based assay buffer supplied with the ROS Detection Cell-Based Assay Kit (DCFDA) (Cayman, Ann Arbor, MI, USA), and 100 μl of ROS staining buffer mixed with 10 μM DCFDA was added and incubated for 90 min at 37 °C protected from light. After incubation, the ROS staining buffer was aspirated, and after further addition of 100 μl of cell-based assay buffer to each well, fluorescence was read on a TECAN Infinite 200 PRO plate reader (Tecan, Morrisville, NC, USA) at excitation and emission wavelengths of 485 and 535 nm, respectively.

### ATP release assays

To evaluate ATP release from hDPSC-K4DT cells, ATP levels were measured in the culture supernatant using a luminescence-based ATP assay kit (TOYO B-Net, Tokyo, Japan). In brief, hDPSC-K4DT cells were seeded in 96-well plates at a density of 5.0 × 10^3^ cells/well, cultured to 70% confluence, and incubated at 37 °C in an osteogenic induction medium for 14 days. Following osteogenic differentiation, HDPSC-K4DT cells were treated with HEMA at 0, 3, and 30 mM for 24 h and ATP levels were measured in cell supernatants using luminescence-based commercial kits (TOYO B-Net, Tokyo, Japan). Luminescence was measured using a microplate reader TECAN infinite 200 PRO with i-control 2.0 software (Tecan, Morrisville, NC, USA). To determine the effect of a free radical scavenger or a TRPA1 inhibitor on HEMA-induced ATP release levels, following osteogenic differentiation, the cells were pretreated with PBN (6 mM) or the TRPA1-selective antagonist HC-030031 (30 or 100 μM, FUJIFILM Wako Chemicals, Miyazaki, Japan) for 1 h, then stimulated with 30 mM HEMA for an additional 24 h before measuring ATP levels as above.

### Statistical analysis

Results are presented as mean ± standard deviation (SD). All the experiments were performed with three or more biological replicates per group. A two-tailed unpaired Student’s t-test was used to evaluate statistical differences between two groups. To evaluate the differences among the three groups, one-way analysis of variance (ANOVA) was used followed by Tukey’s test or Dunnett’s test. *p*-values that were less than 0.05 were considered statistically significant.

## Results

### TRPA1, TRPV1, and TRPV4 expression

**Figure 1:**
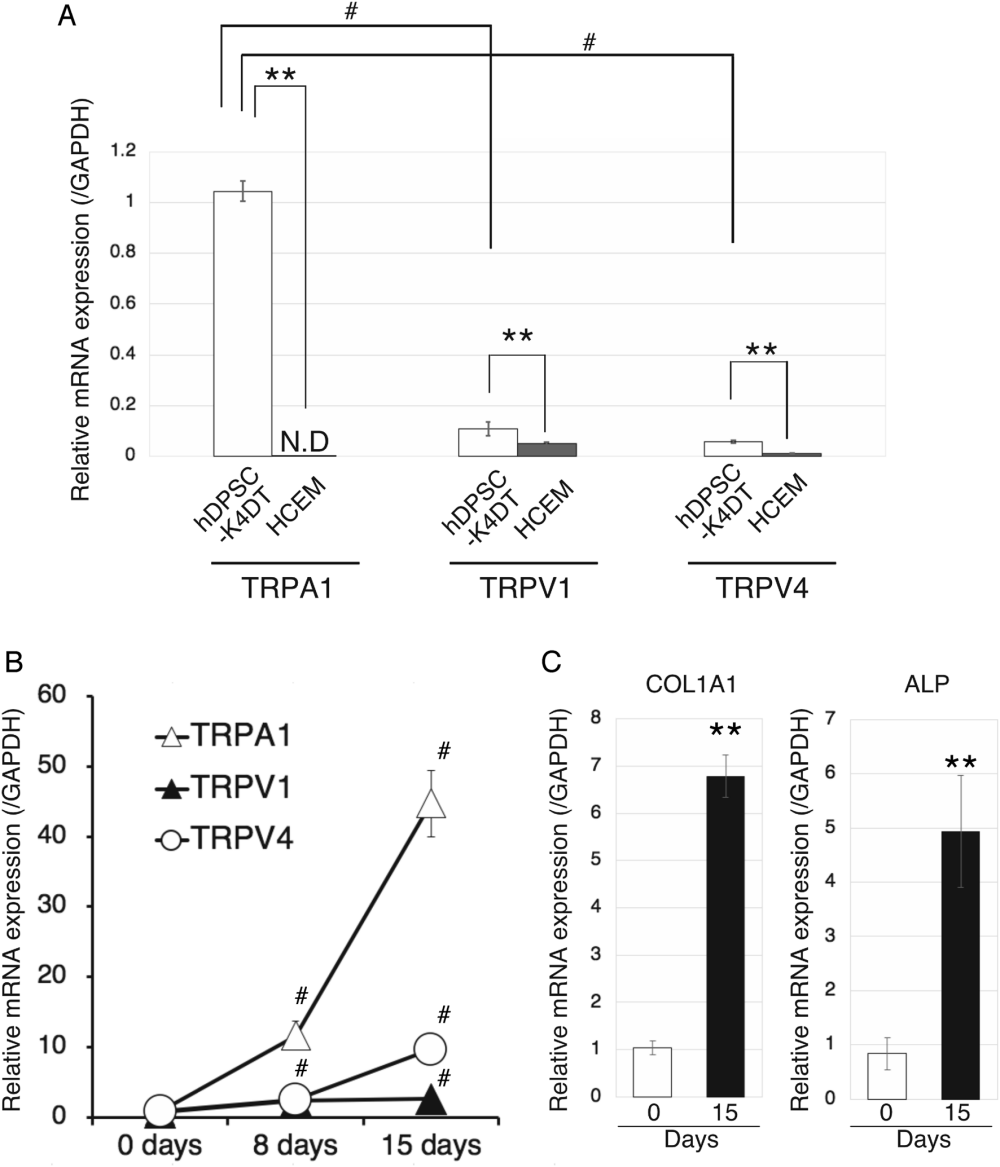
Detection of TRPA1, TRPV1, and TRPV4 in undifferentiated hDPSC-K4DT cells. (**A**) Relative mRNA expression levels of TRPA1, TRPV1, and TRPV4 in undifferentiated hDPSC-K4DT (white bars) and HCEM cells (gray bars), which were analyzed by RT-qPCR. GAPDH was used as the reference gene. The data are presented as the mean ± SD (n = 3). # indicates a significant difference (*P* < 0.05) between TRPA1 and TRPV1, or TRPV4 in undifferentiated hDPSC-K4DT cells by using Dunnett's post hoc test following one-way ANOVA. ** indicates a significant difference (*P* < 0.01) in relative expression levels of TRPA1, TRPV1, and TRPV4 between undifferentiated hDPSC-K4DT and HCEM cells using Student’s *t*-test. (**B**) Osteogenic differentiation enhances TRP channel expression in hDPSC-K4DT cells. Relative mRNA expression levels of TRPA1, TRPV1, and TRPV4 in hDPSC-K4DT cultured with the osteoinduction medium for osteogenic differentiation at 0, 8, and 15 days were measured using qRT-PCR. The data are presented as the mean ± SD (n = 3). Differences were assessed by Dunnett's post hoc test following one-way ANOVA. ^#^*P* < 0.05 compared with 0 day. (**C**) Detection of osteoblastic markers, *COLA1* and *ALP*. Relative mRNA expression levels of *COLA1* and *ALP* at 0 (white bars) and 15 (black bars) days after osteogenic differentiation of hDPSC-K4DT cells were measured using qRT-PCR. The data are presented as the mean ± SD (n = 3). Differences were assessed by Student’s *t*-test. **P < 0.01.

RT-qPCR analysis of the mRNA levels of TRPA1, TRPV1, and TRPV4 in undifferentiated hDPSC-K4DT cells, indicated that those of TRPA1 were the highest among the ROS-sensitive TRP channels (*p* < 0.05, for both TRPV1 and TRPV4, Fig. 1A). In HCEM cells, *TRPA1* mRNA was undetectable and the levels of *TRPV1* and *TRPV4* mRNAs were significantly lower than those in hDPSC-K4DT cells (*p* < 0.01 for both; Fig. 1A). Culture of hDPSC-K4DT cells in the osteoinduction medium, for osteogenic differentiation, resulted in increased mRNA levels of the three TRP channels at 8 and 15 days (*P* < 0.05; Fig. 1B). TRPA1 expression levels at 8 days and 15 days were significantly higher than that of *TRPV1* and *TRPV4* (*p* < 0.05, Fig. 1B). At 15 days, mRNAs levels of the osteoblastic markers collagen type I α 1 chain (*COL1A1*) and alkaline phosphatase (*ALP*) were significantly upregulated compared with undifferentiated hDPSC-K4DT cells (0 day: *p* < 0.01 for both genes; Fig. 1C), indicating successful osteoblastic differentiation of hDPSC-K4DT cells. These results indicated that *TRPA1* may function as the main ROS sensor in undifferentiated and differentiated hDPSC-K4DT cells.

### HEMA toxicity in hDPSC-K4DT

**Figure 2:**
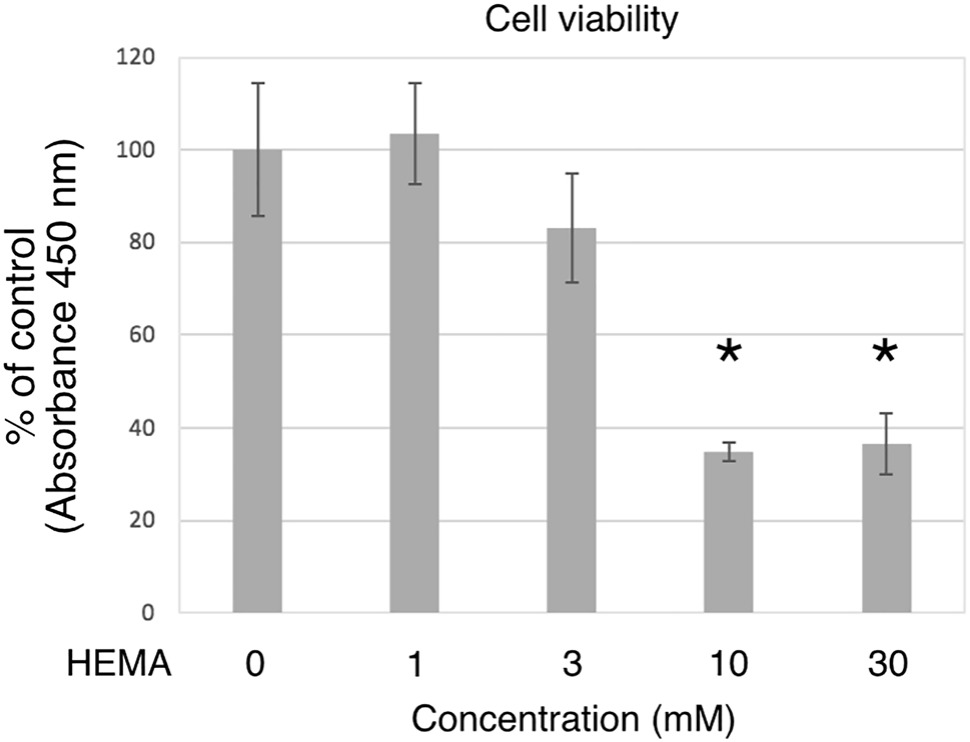
Dose dependent effects of increasing concentrations of HEMA on cell viability measured with the WST-8 assay using Cell Counting Kit-8. Cells were incubated for 24 h with the indicated initial concentrations of HEMA, and cell viability was determined using WST-8. Cell viability is expressed as percentage of the control value, taken as 100%. The data are presented as the mean ± SD (n = 4). Differences were assessed by using Dunnett's post hoc test following one-way ANOVA. *p < 0.05.

Prior to examining the effects of HEMA on hDPSC-K4DT cells, we tested the cytotoxic doses of HEMA on cell viability using the CCK-8 assay. Incubation of undifferentiated hDPSC-K4DT cells with HEMA at 1–30 mM for 24 h, showed that concentrations higher than 10 mM significantly reduced cell viability (*P* < 0.05 for 10 and 30 mM; Fig. 2). Therefore, we used 3 and 30 mM as the non-toxic and toxic doses, respectively, in the following experiments.

### ROS production by the toxic HEMA dose

**Figure 3:**
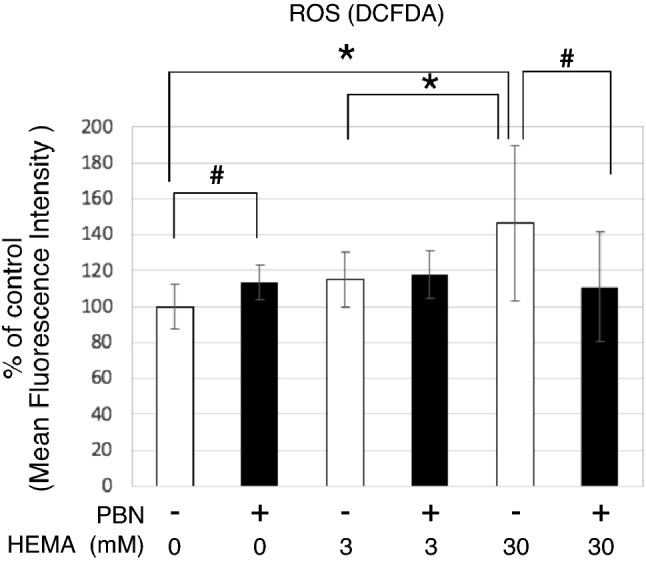
Determination of ROS production of HEMA in osteodifferentiated hDPSC-K4DT cells. Intracellular ROS levels in hDPSC-K4DT at 12 days of culture with the osteoinduction medium for osteogenic differentiation exposed to HEMA in the absence (white bars) or presence (black bars) of 6 mM PBN for 3 h. ROS levels are expressed as percentage of the control value taken as 100%. The data are presented as the mean ± SD (n = 12). Differences were assessed by Student’s *t*-test, ^#^*p* < 0.05 and one-way ANOVA followed by a Tukey test, *p < 0.05.

To evaluate ROS production following HEMA application, osteodifferentiated hDPSC-K4DT cells were treated with 3 and 30 mM HEMA for 3 h, and ROS levels were determined using the ROS Detection Cell-Based Assay Kit (DCFDA). Compared to the control group (0 day), the intracellular ROS levels increased in hDPSC-K4DT cells treated with toxic 30 mM HEMA (*P* < 0.05), but not in cells treated with non-toxic 3 mM HEMA (Fig. 3). Although the free radical scavenger PBN slightly increased ROS levels in HEMA-free conditions, it significantly relieved the overproduction of ROS following treatment with the toxic HEMA dose to the control level (*P* < 0.05; Fig. 3).

### ATP release by the toxic HEMA dose and involvement of TRPA1 activation

**Figure 4:**
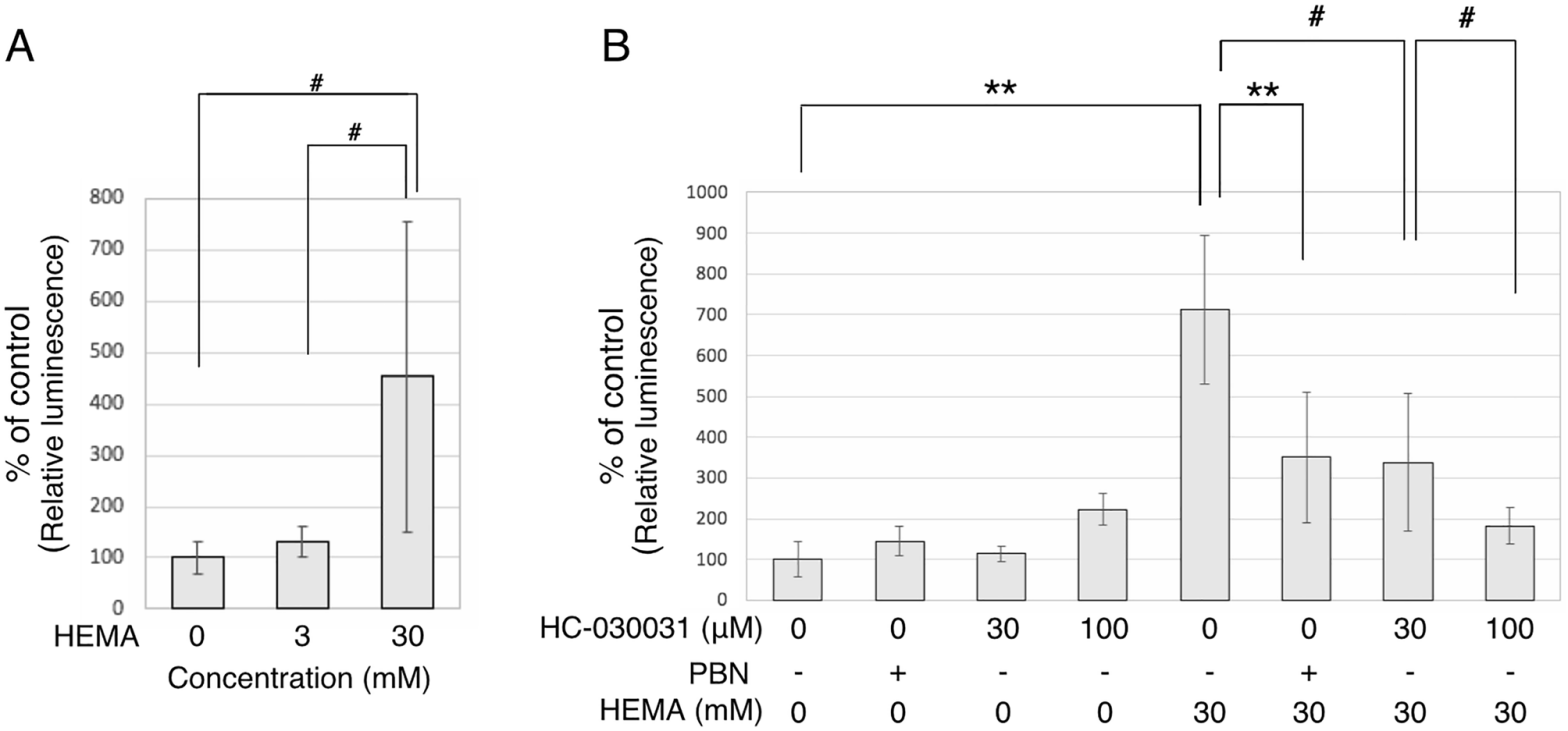
HEMA-induced ATP release from osteodifferentiated hDPSC-K4DT cells. (**A**) Dose dependent effects of HEMA on the extracellular ATP concentration. (**B**) Effect of a free radical scavenger or a TRPA1 antagonist, PBN or HC-030031, respectively, on HEMA-induced ATP release. To assess the HEMA-induced ATP release from the osteodifferentiated hDPSC-K4DT cells, the cells were pretreated for 1 h with 6 mM PBN or the indicated concentrations of HC-030031, and then stimulated with HEMA at 30 mM for an additional 24 h. The ATP levels in the supernatants of cells were measured using a luciferase-based assay. The ATP levels are expressed as percentage of the control value taken as 100%. Bars are means ± SD (n = 6–12). Differences were assessed by one-way ANOVA followed by a Tukey test or Student’s *t*-test. ^#^*p* < 0.05, *p < 0.05, **p < 0.01.

To address the effect of HEMA on ATP release, we measured ATP concentration in culture supernatants following treatment of osteodifferentiated hDPSC-K4DT cells with HEMA for 24 h. As shown in Fig. 3A, 30 mM HEMA significantly increased the extracellular ATP concentration (*P* < 0.05), whereas 3 mM HEMA had no effect (Fig. 4). To determine the involvement of ROS-induced TRPA1 activation, we investigated the effects of PBN and TRPA1 antagonist HC-030031 on HEMA-induced ATP release. As shown in Fig. 4B, pretreatment with both PBN and HC-030031 significantly attenuated the increase in extracellular ATP levels upon treatment with 30 mM HEMA (*P* < 0.01 by PBN and *P* < 0.05 by HC-030031 at 30 and 100 μM), whereas these drugs did not show any effects on HEMA-free conditions. These results suggest that a toxic HEMA dose stimulates ATP release following TRPA1 activation via ROS.

### HO-1 expression after HEMA exposure

**Figure 5:**
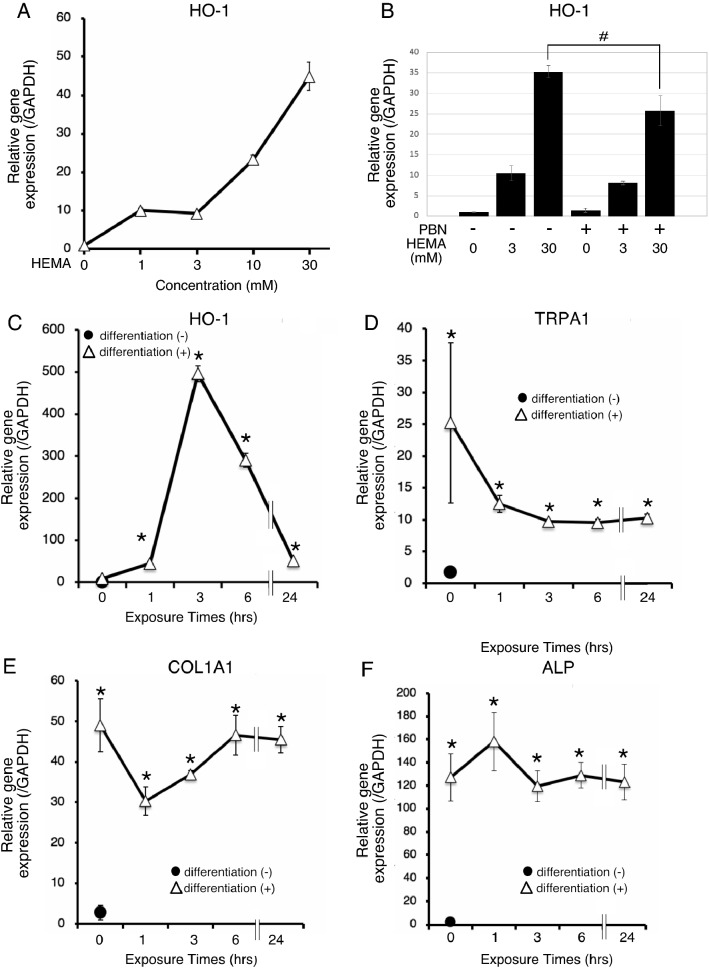
Time- and concentration-dependent effects of HEMA on HO-1 expression levels. (**A**) Concentration-dependent effect of HEMA on HO-1 expression levels. The hDPSC-K4DT cells at 14 days after osteogenic differentiation were incubated for 3 h with several concentrations (0–30 mM) of HEMA, and subjected to RT–qPCR for determining relative mRNA expression levels of HO-1. (**B**) RT-qPCR analysis for HEMA-induced HO-1 after administration of free radical scavengers. We assessed the HEMA-induced HO-1 expression levels in osteodifferentiated hDPSC-K4DT cells pretreated for 1 h with 6 mM PBN followed by 3 h co-incubation with HEMA. The data are presented as the mean ± SD (n = 3). ^#^*P* < 0.05, Student’s t-test. (**C**) Time-dependent effects of HEMA on HO-1 expression levels. The hDPSC-K4DT cells were culture in normal or osteogenic induction medium for 15 days, and then incubated with HEMA (3 mM) for the indicated periods, and RT–qPCR was used to determine relative mRNA expression levels of *HO-1*, (**D**) *TRPA1*, (**E**) *COLA1*, and (**F**) *ALP*. *GAPDH* was used as the reference gene. The data are presented as the mean ± SD (n = 3–6). Differences were assessed by Dunnett's test. *p < 0.05 vs. no differentiation groups.

The detected ROS levels in Fig. 3 is the final output of the intracellular oxidative/anti-oxidative responses. To examine the intracellular anti-oxidant response during HEMA exposure, we investigated the mRNA expression of HO-1, known as a marker of activation of the anti-oxidative Nrf2-ARE pathway, in osteodifferentiated hDPSC-K4DT cells following treatment with HEMA for 3 h using RT-qPCR analysis. Incubation with HEMA increased HO-1 mRNA expression in a dose-dependent manner, even at non-toxic doses (*P* < 0.05; Fig. 5A). Pretreatment with PBN reduced 30 mM HEMA-induced HO-1 upregulation (*P* < 0.05) but did not affect 3 mM HEMA-induced one (Fig. 5B), suggesting oxidative stress-independent activation of the Nrf2-ARE pathway.

PBN-resistant 3 mM HEMA-induced HO-1 upregulation peaked at 3 h and returned to basal levels 24 h after exposure (Fig. 5C). Simultaneously, TRPA1 mRNA expression tended to be reduced 1 h after 3 mM HEMA exposure, although the difference was not statistically significant (Fig. 5D). We examined the possibility of *TRPA1* downregulation along with dedifferentiation of osteodifferentiated hDPSC-K4DT cells, but the osteoblastic markers *COL1A1* and *ALP* did not change throughout the time course of the experiment (Fig. 5E,F).

## Discussion

**Figure 6:**
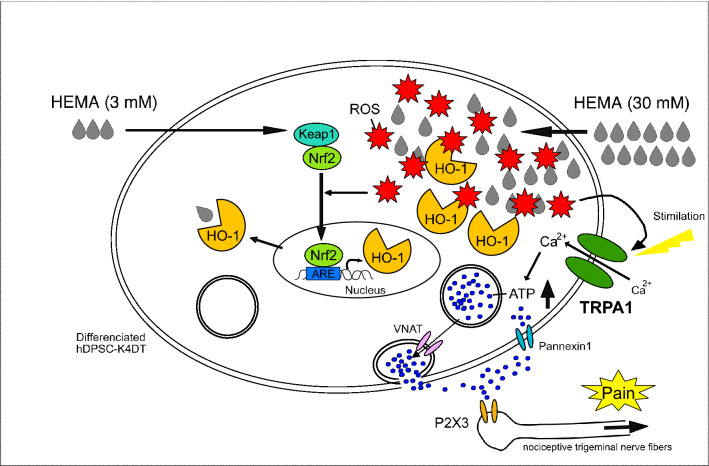
A schematic diagram showing how 30 mM HEMA increases ATP release in hDPSC-K4DT cells. Our findings suggest that the possible activation of TRPA1 by HEMA-induced ROS. Our results also suggest that there are differences in the cellular mechanism of response to HEMA between low concentration (3 mM, see the left hand of figure) and high concentration (30 mM, see the right hand of figure). The induction of HO-1 by 3 mM HEMA may counteract HEMA-induced oxidative stress, which does not cause accumulation of ROS, whereas induction of HO-1 by 30 mM HEMA cannot counteract HEMA-induced sever oxidative stress, which causes ROS accumulation. The following the increase in the extracellular ATP levels suggests TRPA1 activation by ROS stimulation and induction of ATP release, which might be involved in pain conduction and sensation.

In this study, we demonstrated that the overproduction of ROS caused by high-dose of HEMA activates the TRPA1 channel and stimulates ATP release (Fig. 6). High concentrations of HEMA (30 mM) increased the expression of the oxidative stress marker HO-1 and the production of ROS. Furthermore, ROS scavengers decreased their levels induced by 30 mM HEMA, but not by 3 mM HEMA. These results and those from previous studies^11,12^ support the view that the activation of the Nrf2-ARE/HO-1 transcriptional pathway by HEMA at low concentrations is mainly independent of oxidative stress and is suggestive of direct binding to Keap-1.

Activation of the Nrf2-ARE/HO-1 transcriptional pathway in differentiated hDPSC-K4DT cells exposed to low concentrations of HEMA (3 mM) may increase the levels of proteins critical for detoxification, such as HO-1, and eliminate ROS, indicating that HEMA at low concentrations does not cause accumulation of ROS. In contrast, in cells exposed to high concentrations of HEMA (30 mM), induction of HO-1 cannot counteract HEMA-induced severe oxidative stress, which causes ROS accumulation. The increase in extracellular ATP levels in the cells suggested the possibility of *TRPA1* activation by ROS stimulation. Others demonstrated that TRPA1 antagonist HC-030031 suppressed H_2_O_2_ production-induced intracellular ROS, ATP release, and the mRNA and protein levels of PANX1^32^. These finding suggest that ATP is released via PANX1 in response to TRPA1 channel activation by the H_2_O_2_-induced ROS stimulation. Our results indicated that TRPA1 antagonist HC-030031 also decreased the HEMA-induced intracellular ROS and ATP release. The HEMA-induced ATP release, probably via PANX1 in response to TRPA1 channel activation as well with previous reports^33^. Secreted ATP has been shown to activate ionotropic P2X3 receptors on nociceptive trigeminal nerve fibers, which mediate sensory transduction to the brain and induce dental pain (Fig. 6), according to a previous study^33^. There were differences in the cellular response and defense mechanisms against oxidative stress induced by HEMA at low and high concentrations. From this accumulative side supportive evidence, we explain the different observations that HO-1 and ROS induced by 30 mM HEMA but not by 3 mM HEMA were counteracted by ROS scavengers.

Recent studies in rat incisor models of tooth bleaching have suggested that strong oxidative stress following treatment with high-dose H_2_O_2_ induced ROS-mediated cytotoxicity in DPSCs, which could affect the hyperalgesia of tooth by regulating *TRPA1* expression, intracellular Ca^2^^+^ concentration, and ATP release into the extracellular space^32,34^. Moreover, oxidative stress in the periodontal tissue and dental pulp following orthodontic force is suggested to activate and/or mechanically sensitize *TRPA1* on nociceptive fibers, resulting in orthodontic nociception^35^. Our data showed that HEMA-induced oxidative stress with the accumulation of ROS activates TRPA1 channels, consistent with the fact that *TRPA1* can be activated by multiple products of oxidative stress^19^. This study provides new insights into cellular responses to HEMA exposure, which may be involved in the pain response through *TRPA1* activation^19^.

In this study, the previously established immortalized human DPSCs, hDPSC-K4DT cells^30^. The previous study has shown that the hDPSC-K4DT has the ability to differentiate into the osteogenic by Alizarin Red S staining and immunostaining with anti-osteocalcin antibody at later passages more than that in this study. The hDPSC-K4DT cells exhibited a time-dependent increase in the mRNA expression of *COL1A1* and *ALP*, markers of differentiation in osteogenic cells, suggesting the maintenance of osteogenic differentiation potential. Our previous study and this study demonstrated that hDPSC-K4DT cells retained the original differentiation abilities of human dental pulp cells. Furthermore, consistent with previous reports^23,36^, *TRPA1* was highly expressed in hDPSC-K4DT cells, and *TRPA1* expression was elevated along with their osteogenic differentiation. These results indicate that hDPSC-K4DT cells are a useful in vitro tool for studying the cytotoxicity of HEMA and can be used for the evaluation of the relationship between cytotoxicity and TRPA1 channels. Further elucidation of the functional role of *TRPA1* in DPSCs is necessary to understand the transmission of pain sensations.

In summary, the present study suggests that high-dose but not low-dose HEMA activates TRPA1 channels through ROS generation in immortalized human DPSC. This study contributes to the understanding of the underlying molecular mechanism of dental pain conduction via *TRPA1* activation in response to resin monomers and biomaterials.

## Data Availability

All relevant data are within the paper.
